# Transcutaneous electrical acupoint stimulation alleviates cerebral ischemic injury through the TLR4/MyD88/NF-κ B pathway

**DOI:** 10.3389/fncel.2023.1343842

**Published:** 2024-01-11

**Authors:** Linyu Wu, Zixuan Tan, Lei Su, Fang Dong, Guangyu Xu, Feng Zhang

**Affiliations:** ^1^Department of Rehabilitation Medicine, The Third Hospital of Hebei Medical University, Shijiazhuang, People’s Republic of China; ^2^Department of Radiotherapy, Affiliated Hospital of Hebei University, Baoding, China; ^3^Department of Clinical Laboratory Medicine, The Third Hospital of Hebei Medical University, Shijiazhuang, People’s Republic of China

**Keywords:** ischemic stroke, transcutaneous electrical acupoint stimulation, TLR4, inflammation, cell death, microglia activation

## Abstract

This study was to explore whether transcutaneous electrical acupoint stimulation (TEAS) treatment could mediate inflammation, apoptosis, and pyroptosis of neuronal cells and microglia activation through the TLR4/MyD88/NF-κB pathway in the early stage of ischemic stroke. TEAS treatment at Baihui (GV20) and Hegu (LI4) acupoints of the affected limb was administered at 24, 48, and 72 h following middle cerebral artery occlusion/reperfusion (MCAO/R), with lasting for 30 min each time. Neurological impairment scores were assessed 2 h and 72 h after ischemia/reperfusion (I/R). TTC staining was used to evaluate the volume of brain infarction. The histopathologic changes of hippocampus were detected by H&E staining. WB analysis was performed to assess the levels of TLR4, MyD88, p-NF-κB p65/NF-κB p65, and inflammation, apoptosis, pyroptosis-related proteins. TLR4 expression was measured using immunohistochemistry. The expression of inflammation-related proteins was also measured using ELISA. Immunofluorescence was used to detect the expression level of Iba1. Our findings demonstrated that TEAS intervention after I/R improved neurological function, reduced the volume of brain infarction, and mitigated pathological damage. Moreover, TEAS reduced the levels of TLR4, MyD88, p-NF-κB p65/NF-κB p65, TNF-α, IL-6, Bax, NLRP3, cleaved caspase-1/pro caspase-1, IL-1β, IL-18, GSDMD, and Iba1 while enhancing Bcl-2 expression. Moreover, the protective effects of TEAS could be counteracted by lipopolysaccharide (LPS, a TLR4 agonist). In conclusion, TEAS can reduce cerebral damage and suppress inflammation, cell death, and microglia activation after ischemic stroke via inhibiting the TLR4/MyD88/NF-κB pathway.

## Introduction

Stroke is one of the major reasons for serious death and disability all around the world, which is characterized by elevated levels of morbidity, relapse, fatality, and impairment (Huang et al., 2021). Due to inadequate blood circulation to the brain, stroke can be classified into two primary types: hemorrhagic stroke and ischemic stroke (Jun-Long et al., 2018). Among them, 84.4% of the stroke patients are ischemic stroke GBD 2016 Stroke Collaborators (2019). Currently, the administration of thrombolytic therapy within 4 h of the start of symptoms is the most effective treatment for ischemic stroke (Herpich and Rincon, 2020). Nevertheless, the utilization of thrombolysis is restricted due to the limited timeframe for treatment and the potential hazard of bleeding (Jaffer et al., 2011). Furthermore, thrombolytic treatment not only reinstates the circulation of blood, but it can also lead to inflammation and apoptosis, thereby exacerbating brain ischemia/reperfusion (I/R) damage (Xu et al., 2021; Zhang and Cui, 2021). Thus, how to attenuate I/R damage has become a crucial problem in cerebral ischemia.

Toll-like receptor 4 (TLR4), as the first recognized Toll-like receptors (TLRs) in human, encompasses a leucine-rich repeat in extracellular area and a Toll/IL-1 receptor homology (TIR) domain in intracellular area (Beutler et al., 2006). Mutual influence exists between the TIR domain and myeloid differentiation factor 88 (MyD88) (Kawai et al., 2001). When TLR4/MyD88 is activated, it moves nuclear factor kappa-B (NF-κB) from the cytoplasm to the nucleus, resulting in the generation of inflammatory chemokines such as tumor necrosis factor-α (TNF-α), Interleukin-1β (IL-1β), and IL-6. This, in turn, initiates an inflammatory reaction (Barton and Medzhitov, 2003). Moreover, TLR4 is activated significantly in the brain I/R damage model, and TLR4 inhibitors are capable of decreasing cerebral infarct volume and reducing the release of inflammatory factors (Hua et al., 2015). Electroacupuncture (EA) inhibited the inflammatory response in microglia via suppressing the TLR4/NF-κB pathway in MCAO rats (Han et al., 2015). Hence, the TLR4/MyD88/NF-κB signaling pathway has the ability to control inflammation in neuronal cells following ischemic stroke.

Nod-like receptor protein 3 (NLRP3) inflammasome, which is extensively researched among the NLRP proteins, has the ability to regulate cellular inflammation and pyroptosis (Chen and Xu, 2022). The TLR4/NF-κB pathway has a strong connection to the activation of NLRP3 inflammasome (El-Sisi et al., 2021). Through the inhibition of the TLR4/NF-κB signaling pathway, meisoindigo effectively reduced cerebral ischemia injury by preventing NLRP3 inflammasome activation and the polarization of microglia M1 (Ye et al., 2019). Thus, TLR4/MyD88/NF-κB signaling pathway exerts vital importance on regulating NLRP3 activation, neuronal pyroptosis and microglia activation after cerebral ischemia.

Transcutaneous electrical acupoint stimulation is an emerging treatment that brings together the benefits of transcutaneous electrical nerve stimulation (TENS) and conventional acupuncture (Szmit et al., 2023). Accumulating evidence indicates that TEAS is capable of exerting therapeutic benefits in stroke rehabilitation. For example, the combination of TEAS with conventional rehabilitation programme could improve the strength of wrist extension and hand grip of the hemiplegic side in stroke patients (Wang et al., 2023). Moreover, TEAS therapy at lower leg acupoints after stroke could obviously reduce ankle plantar flexor cramp and increase dorsi flexor strength in clinic (Yan and Hui-Chan, 2009). Therefore, TEAS can improve various functional disorders after stroke. Currently, TEAS is primarily utilized during the subacute and later stages after a stroke. However, its usage in the early phase after a stroke is severely limited due to the lack of widespread recognition regarding its effectiveness and the absence of clear understanding regarding its effectiveness and exact mechanisms. Hence, it is of great clinical importance to investigate the pathophysiological mechanisms of TEAS in order to provide solid evidence for its clinical application in the early stage of ischemic stroke.

The current research validated the advantageous function of TEAS during the initial stage after I/R and examined the neuroprotective mechanisms of TEAS (including reducing neuronal inflammation, apoptosis, and pyroptosis, as well as suppressing microglia activation) through the TLR4/MyD88/NF-κB pathway after ischemic stroke.

## Materials and methods

### Experimental animals and groups

Male Sprague-Dawley (SD) rats weighing between 250 and 280 g were obtained from Beijing Huafukang Biotechnology Co., LTD. The rats were housed in a standard animal room with a temperature of 22 ± 2°C, humidity ranging from 60 to 70%, and a 12-h light-dark cycle. They were provided with sufficient food and water. Approval for the experimental procedure was granted by Laboratory Animal Ethical and Welfare Committee of Hebei Medical University.

**FIGURE 1:**
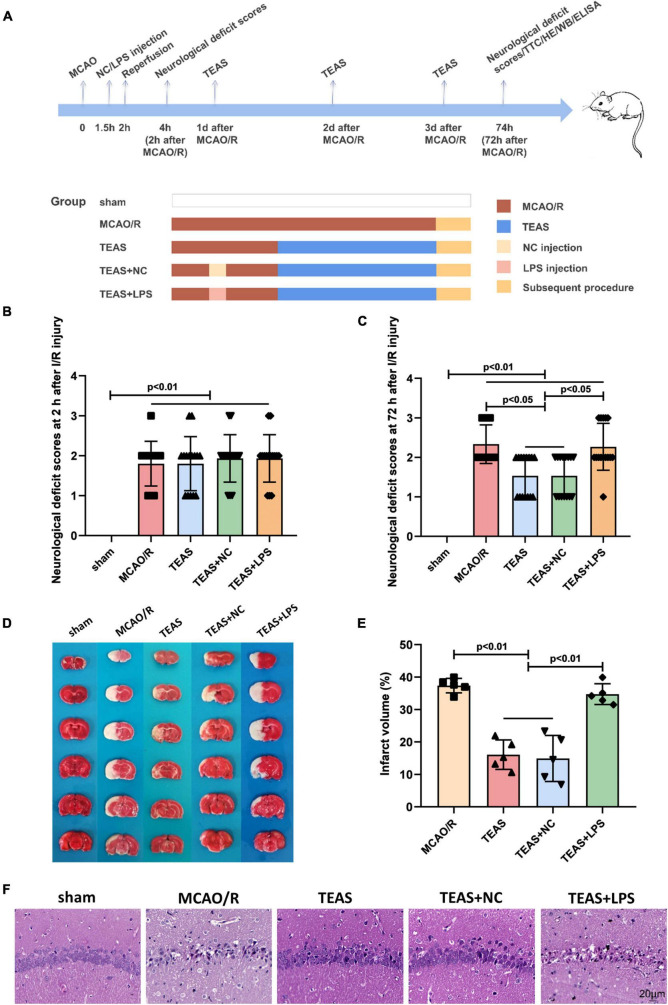
The pathological changes following I/R within a 72-h period. **(A)** The experimental scheme in various time points. **(B)** Scores of neurological deficits observed 2 h after I/R. **(C)** Neurological impairment assessments 72 h post I/R. **(D)** Staining with TTC in various groups. **(E)** According to statistical analysis, the proportion of brain infarct volume was indicated. **(F)** Staining of rats in various groups using the H&E. Scale bar = 20 μm.

The rats were divided into five groups at random, each consisting of 25 rats, as depicted in Figure 1A. The groups were as follows: (i) Sham group, where the rats underwent surgery without occlusion of the MCA; (ii) MCAO/R group, where the left MCA was blocked for 2 h and then reopened; (iii) TEAS group, where electrical stimulation was administered for 30 min daily after MCAO, at 24 h, 48 h, and 72 h after the ischemia/reperfusion; (iv) TEAS + NC group, where NC (saline, a non-specific control of LPS) was injected intraperitoneally 1.5 h after MCAO. The remaining procedures mirrored those of TEAS group. (v) TEAS + lipopolysaccharide (LPS) group, where the TLR4 agonist LPS (Han et al., 2016) (1 mg/kg, Beijing Kulaibo Technology Co., Ltd., Beijing, China) was dissolved in normal saline as directed. The rats received an intraperitoneal injection of the solution 1.5 h after MCAO. Other interventions were the same as TEAS group.

### Establishment of MCAO rat model

The occlusion of the left middle cerebral artery (MCA) led to the establishment of the MCAO/R model. In short, we inserted a surgical nylon suture (Beijing Xinong Co., Ltd., Beijing, China) from the left common carotid artery (CCA) to the internal carotid artery (ICA) to block the MCA. The operation was detailed in our previous publications (Xing et al., 2018a).

### TEAS intervention

Transcutaneous electrical acupoint stimulation therapy was performed at 24 h after MCAO/R by using a low frequency electrical current pulse therapeutic equipment (Model G6805-2A; Shanghai Huayi Co., China), once a day, 30 min each time for 3 consecutive days. Rats underwent anesthetization with 3% pentobarbital sodium before TEAS stimulation. In the right limb, two electrodes measuring 5 mm × 5 mm were securely placed at the acupoints of Baihui (GV20) and Hegu (LI4). The intervention settings were configured with a dense-disperse wave frequency of either 4 or 20 Hz and an intensity of 3–4 mA, which was adjusted based on the muscle twitch threshold. At 72 h following MCAO/R, the rats in every group were beheaded for the subsequent experiment.

### Neurological deficit scores evaluation

The neurological deficits scores of each rat (125 rats in total) were rated blindly at 2 h or 72 h following I/R damage in a blind method. The detailed information is described in our previous articles (Xing et al., 2018a).

### 2,3,5-Triphenyltetrazolium Chloride (TTC) staining

After 72 h of reperfusion, the rats (*n* = 5/group) were promptly sacrificed and then frozen at −20°C for a duration of 15 min. Then cut the brain into six slices with a thickness of 2 mm per slice. The slices were immersed in a 2% TTC solution (Solarbio, China). The details for procedures are demonstrated in the section of Supplementary materials.

### Histopathological analysis

Five samples of brain tissue per group were embedded in paraffin, followed by staining the slices with hematoxylin and eosin (H&E). The histopathological alterations were captured with a 400 × magnification microscope (Olympus, Japan).

### Western blot (WB) analysis

Western blot was used to detect the levels of proteins in the TLR4/MyD88/NF-kB pathway and proteins related to inflammation, apoptosis, and pyroptosis, including TNF-α (1:500, AF7014, Affinity), IL-6 (1:2000, DF6087, Affinity), Bcl-2 (1:1000, ARG55188, arigo), Bax (1:5000, #60267-1-Ig, proteintech), NLRP3 (1:1000, #19771-1-Ig, proteintech), cleaved caspase-1 (1:1000, #AF4005, Affinity), pro caspase-1 (1:1000, ET1608-69, Hangzhou Hua’an Biotechnology Co., Ltd.), IL-1β (1:2000, AF5103, Affinity), IL-18 (1:2000, #60070-1-Ig, proteintech), GSDMD (1:500, #20770-1-AP, proteintech), TLR4 (1:1000, AF7017, Affinity), MyD88 (1:1000, AF5195, Affinity), p-NF-κB p65 (1:1000, #3033), and NF-κB p65 (1:1000, #8242). Rats (*n* = 5 per group) were sacrificed and the target protein was detected. The injured hippocampus was collected and hippocampal tissues were lysed. The details for procedures are demonstrated in the section of Supplementary materials.

### Immunohistochemistry (IHC) analysis

Five rats per group were decapitated under anesthesia, and their brains were fixed in 4% paraformaldehyde for 24 h. Paraffin-embedded tissues were prepared. The details for procedures are demonstrated in the section of Supplementary materials.

### ELISA assay

Hippocampal tissues of rats (5 in each group) and homogenized in lysis buffer. The supernatants were gathered and the levels of TNF-α, IL-6 (Beijing Sizhengbai Biotechnology Co., Ltd, China), and IL-18 (Xinbosheng Biotechnology Co., Ltd, China) were assessed using the standard ELISA kit, following the guidelines provided by the manufacturer. The microplate reader from Thermo Fisher Scientific, America was used to detect the optical density (OD) value at 450 nm, and the data was calculated using the standard provided in the kits.

### Immunofluorescence (IF) staining

Rats (*n* = 5 per group) were decapitated under anesthesia. Immediately, the brain was preserved using a 4% solution of paraformaldehyde. Following a 24-h fixation period, the brains were encased in paraffin blocks. Coronal sections of 4 μm thickness were obtained by cutting the paraffin blocks. The details for procedures are demonstrated in the section of Supplementary materials.

### Statistical analysis

SPSS 26.0 was used to implement all the data. The mean ± SD was used to present all values. Analysis of variance (ANOVA) was used to analyze data from various groups, followed by least significant difference (LSD) test for two groups comparisons. The five groups were analyzed for neurological deficits scores using a non-parametric test. *P* < 0.05 was deemed statistically significant.

## Results

### TEAS alleviates neurological behavioral scores, infarct volumes, and pathological injury in MCAO/R rats

Neurological behavioral scores were evaluated at 2 h and 72 h following MCAO/R damage. In Figure 1B, the rats of sham group did not display any noticeable neurological deficits (*P* < 0.01), whereas rats in the remaining groups exhibited evident neurological impairments. However, there were no significant variations in neurological scores among the other groups after 2 h of I/R. Nevertheless, as shown in Figure 1C, after 72 h of I/R, TEAS and TEAS + NC groups exhibited significantly reduced neurological behavioral scores in comparison with MCAO/R group (*P* < 0.05). The rats in TEAS + LPS group had significantly higher neurological deficits scores (*P* < 0.05) in comparison with both TEAS and TEAS + NC group.

The size of the infarction was assessed 72 h following reperfusion. In Figure 1D, it can be observed that the rats in sham group had no infarct volume, whereas the rats in other groups displayed a considerable infarct area. The infarct volume of rats in MCAO/R group was increased significantly in comparison with both TEAS (*P* < 0.01) and TEAS + NC group (*P* < 0.01). The infarct volume of rats in TEAS + LPS group was increased (*P* < 0.01) in comparison with both TEAS group and TEAS + NC group, as depicted in Figure 1E.

Figure 1F illustrates the assessment of brain tissue damage through HE staining to determine pathological injury. The examination of brain tissue indicated that the neuronal cells in the hippocampus of the control group were organized in a tidy manner, displaying a typical cellular structure, distinct boundaries, and consistent staining of both the nucleus and substances between cells. The organization of neuronal cells in MCAO/R group was disrupted, showing increased gaps between cells, reduced size of the nucleus and cell body, and the presence of vacuolar degeneration. Following TEAS therapy, the histopathological abnormalities were improved in both TEAS and TEAS + NC cohorts. Nevertheless, the severity of histopathological abnormalities in TEAS + LPS group was worse in comparison with both TEAS and TEAS + NC groups.

### TEAS reduces the release of inflammatory factors in MCAO/R rats

**FIGURE 2:**
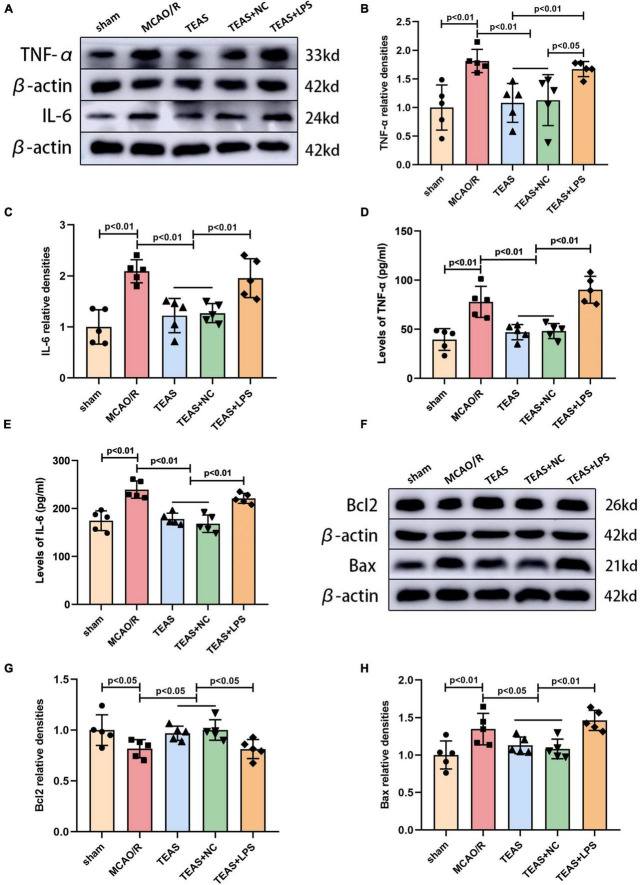
Effects of TEAS on inflammation-associated and apoptosis-associated proteins after I/R. **(A)** Representative western belts of TNF-α and IL-6. **(B,C)** Bar graph showed the difference of TNF-α and IL-6 expression. **(D,E)** ELISA analysis showed the difference of TNF-α and IL-6. **(F)** Representative western blot bands of the levels of Bcl-2 and Bax. **(G,H)** Quantitative analysis suggested the different levels of Bcl-2 and Bax.

In stroke, the excessive inflammatory response can induce secondary neurodegeneration, thus exacerbating cell death. Thus, mitigating the exaggerated inflammatory response can relieve brain damage following ischemia. To investigate if TEAS could suppress cellular inflammation caused by MCAO/R, WB and ELISA techniques were used to measure the levels of two inflammatory cytokines in the hippocampus, as shown in Figure 2. Figures 2B, D clearly show that MCAO/R group exhibited a noticeable increase in TNF-α expression in comparison with sham group (*P* < 0.01). Following TEAS treatment, the levels of TNF-α were reduced in both TEAS group (*P* < 0.01) and TEAS + NC group (*P* < 0.01) in comparison with MCAO/R group. The TNF-α expression in TEAS + LPS group was obviously elevated in comparison with both TEAS (*P* < 0.01) and TEAS + NC group (*P* < 0.05) in Figure 2B. Furthermore, the TNF-α expression in TEAS + LPS group was obviously elevated in comparison with both TEAS (*P* < 0.01) and TEAS + NC group (*P* < 0.01) in Figure 2D. According to the data shown in Figures 2C, E, the level of IL-6 in MCAO/R group exhibited an obvious increase (*P* < 0.01) in comparison with sham group. Following TEAS therapy, the level of IL-6 in both TEAS and TEAS + NC groups exhibited a significant reduction (*P* < 0.01). Furthermore, the IL-6 expression exhibited a noticeable increase in TEAS + LPS group (*P* < 0.01) in comparison with both TEAS and TEAS + NC groups.

### TEAS treatment decreases apoptosis-related proteins in MCAO/R rats

Western blot analysis revealed the presence of proteins associated with apoptosis, as depicted in Figure 2F. According to Figure 2G, the B-cell lymphoma-2 (Bcl2) expression in MCAO/R group was remarkably reduced in comparison with sham group (*P* < 0.05). TEAS therapy significantly reversed the decrease of Bcl2 in both TEAS group (*P* < 0.05) and TEAS + NC group (*P* < 0.05) in comparison with MCAO/R group. Furthermore, when compared to TEAS group (*P* < 0.05) and TEAS + NC group (*P* < 0.05), there was an obvious reduction in the expression of Bcl2 in TEAS + LPS group. Figure 2H demonstrates that Bcl-2 associated X protein (Bax) exhibited a significant increase in MCAO/R group (*P* < 0.01) in comparison with sham group. However, this alteration was effectively reversed in both TEAS group (*P* < 0.05) and TEAS + NC group (*P* < 0.05). Furthermore, there was a higher expression of Bax in TEAS + LPS group in comparison with both TEAS group (*P* < 0.01) and TEAS + NC group (*P* < 0.01).

### TEAS inhibits the activation of NLRP3-mediated pyroptosis in MCAO/R rats

**FIGURE 3:**
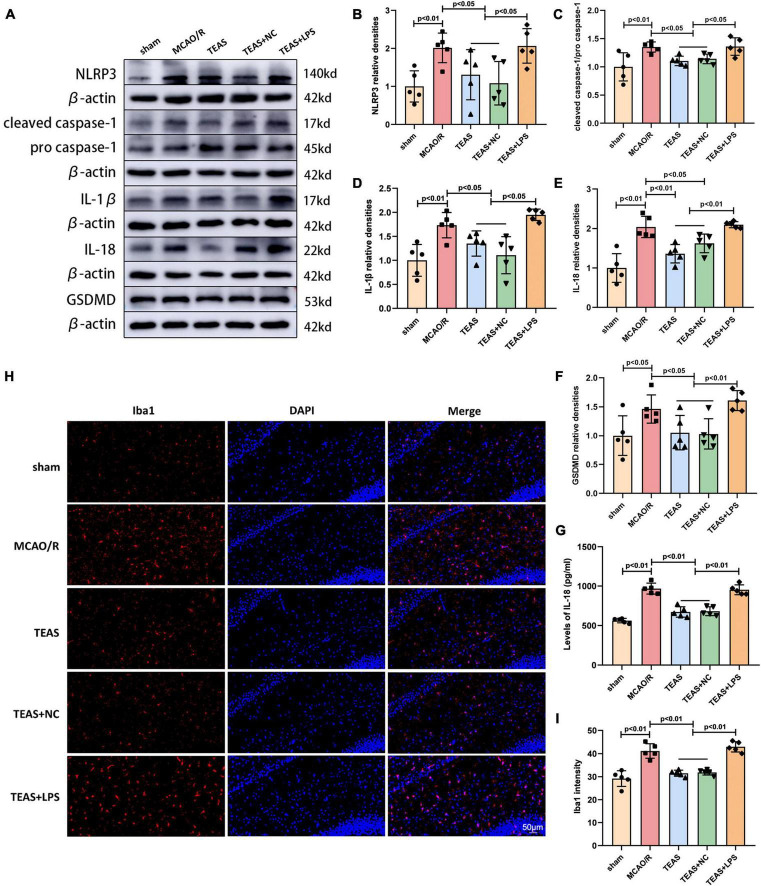
Effect of TEAS treatment on pyroptosis-related proteins and microglia activation in ischemic stroke rats. **(A)** Representative western blot belts for NLRP3, cleaved caspase-1/pro caspase-1, IL-1β, IL-18 and GSDMD. **(B–F)** Statistical analysis of the difference of NLRP3, cleaved caspase-1/pro caspase-1, IL-1β, IL-18, and GSDMD. **(G)** ELISA assay showed the expression of IL-18. **(H)** Immunofluorescence analysis showed the expression difference of Iba1 in the ipsilateral hippocampus of the rats. **(I)** Statistical analysis of the Iba1 expression. Scale bar = 50 μm.

In order to investigate if TEAS can regulate NLRP3 inflammasome activation and neuronal pyroptosis via the TLR4 pathway, WB analysis was employed to measure the levels of NLRP3, cleaved caspase-1/pro caspase-1, IL-1β, IL-18, and Gasdermin D (GSDMD), as depicted in Figure 3A. The levels of NLRP3 (*P* < 0.01, Figure 3B), cleaved caspase-1/pro caspase-1 (*P* < 0.01, Figure 3C), IL-1β (*P* < 0.01, Figure 3D), IL-18 (*P* < 0.01, Figure 3E), and GSDMD (*P* < 0.05, Figure 3F) were increased in MCAO/R group in comparison with sham group. Following the TEAS intervention, TEAS group exhibited reduced levels of NLRP3 (*P* < 0.05), cleaved caspase-1/pro caspase-1 (*P* < 0.05), IL-1β (*P* < 0.05), IL-18 (*P* < 0.01), and GSDMD (*P* < 0.05) in comparison with MCAO/R group. TEAS + NC group showed decreased levels of NLRP3 (*P* < 0.05), cleaved caspase-1/pro caspase-1 (*P* < 0.05), IL-1β (*P* < 0.05), IL-18 (*P* < 0.05), and GSDMD (P < 0.05) in comparison with MCAO/R group. Furthermore, TEAS + LPS group exhibited significantly elevated levels of NLRP3 (*P* < 0.05), cleaved caspase-1/pro caspase-1 (*P* < 0.05), IL-1β (*P* < 0.05), IL-18 (*P* < 0.01), and GSDMD (*P* < 0.01) compared to TEAS and TEAS + NC groups. According to Figure 3G, the ELISA results also indicated a higher IL-18 level of MCAO/R group in comparison with sham group (*P* < 0.01). Following the TEAS treatment, there was a remarkable decrease (*P* < 0.01) in the level of IL-18 expression. Nevertheless, in comparison with TEAS group and TEAS + NC group, the level of IL-18 exhibited an obvious elevation in TEAS + LPS group (*P* < 0.01).

### TEAS treatment inhibits microglia activation in MCAO/R rats

In order to confirm the effect of TEAS therapy on the activation of microglia following cerebral ischemia, the immunofluorescence technique was utilized to evaluate the expression of Iba1, a signature molecule of activated microglia. Figures 3H, I showed that the expression of Iba1 was higher in MCAO/R group in comparison with sham group (*P* < 0.01). Following TEAS treatment, there was a notable reduction in Iba1 expression observed in both TEAS and TEAS + NC groups (*P* < 0.01). Nevertheless, the levels of Iba1 were increased in TEAS + LPS group in comparison with TEAS and TEAS + NC groups (*P* < 0.01).

### TEAS intervention inhibits the TLR4/MyD88/NF-kB pathway in MCAO/R rats

**FIGURE 4:**
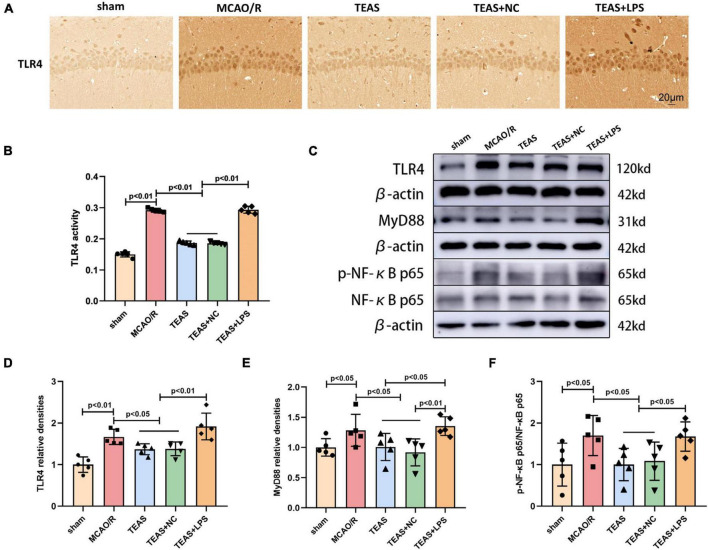
The effect of TEAS therapy on the key proteins of TLR4/MyD88/NF-κB in rats following brain ischemia. **(A,B)** IHC staining and a bar diagram depicted the expression of TLR4 in various groups. Scale bar = 20 μm. **(C)** Exemplary WB bands depicted TLR4, MyD88, p-NF-κB p65, and NF-κB p65. **(D–F)** The bar chart indicated variations in the levels of TLR4, MyD88, and p-NF-κB p65/NF-κB p65.

The TLR4 receptor and related signaling pathway exert vital effects for development of ischemic stroke. Therefore, our hypothesis suggests that TEAS might have a positive impact on MCAO/R via the modulation of the TLR4/MyD88/NF-kB pathway. TLR4 levels were evaluated using IHC. Figures 4A, B demonstrate that MCAO/R group exhibited elevated TLR4 levels in comparison with sham group (*P* < 0.01). Nevertheless, the TEAS therapy significantly reversed this alteration in both TEAS and TEAS + NC groups (*P* < 0.01). Furthermore, the level of TLR4 was remarkably higher in TEAS + LPS group in comparison with TEAS and TEAS + NC groups (*P* < 0.01).

The WB technique was used to determine the levels of TLR4, MyD88, phospho-NF-κB p65 (p-NF-kB p65), and NF-kB p65, as shown in Figure 4C. After MCAO/R, the levels of TLR4 (P < 0.01, Figure 4D), MyD88 (P < 0.05, Figure 4E), and p-NF-kB p65/NF-kB p65 (*P* < 0.05, Figure 4F) showed an increase when in comparison with sham group, as indicated by the results. The levels of the aforementioned proteins (*P* < 0.05) were decreased in TEAS group and TEAS + NC group in comparison with MCAO/R group. Nevertheless, the protein levels of TLR4 (*P* < 0.01), MyD88 (*P* < 0.05), and p-NF-kB p65/NF-kB p65 (*P* < 0.05) exhibited an obvious promotion in TEAS + LPS group in comparison with TEAS group. Compared with TEAS + NC group, TEAS + LPS group showed elevated levels of TLR4 (*P* < 0.01), MyD88 (*P* < 0.01), and p-NF-kB p65/NF-kB p65 (*P* < 0.05).

## Discussion

At present, TEAS are mainly used in the middle and late stage following stroke. Nevertheless, its utilization is restricted during the initial stage of stroke due to the lack of understanding regarding its mechanisms. Hence, our investigation examined if TEAS exhibited defensive properties and further explored the associated mechanisms during the initial stage of ischemic stroke. In previous studies, EA played a protective role in ischemic stroke by regulating apoptosis, inflammation, pyroptosis and other pathological processes (Xing et al., 2018b; Tang et al., 2023). EA treatment at Baihui and Hegu acupoints has been shown to mitigate cerebral ischemic injury (Jiang et al., 2017). Therefore, we selected these two acupoints for TEAS treatment to explore whether TEAS played a protective role in stroke. The findings from our study indicated that applying TEAS therapy at the Baihui and Hegu acupoints was capable of reducing neurological dysfunction following cerebral ischemia, as well as suppressing the activation of microglia and the inflammation, apoptosis, and pyroptosis of neuronal cells. These effects were achieved by suppressing the TLR4/MyD88/NF-κB pathway (Supplementary Figure 1). Consequently, our results provided theoretical evidence for TEAS clinical application to treat acute ischemic stroke.

Ischemic injury stimulates the generation of inflammatory mediators, for instance TNF-α and IL-1β. Meanwhile, ischemia diminishes the release of anti-inflammatory substances, for instance IL-10, while enhancing inflammation, ultimately causing brain injury and neuronal demise following an ischemic stroke (Petrovic-Djergovic et al., 2016). More importantly, revascularization of cerebral blood flow further exacerbates the inflammation triggered by cerebral ischemia (Mizuma and Yenari, 2017). Therefore, suppressing inflammation could be a vital approach for treating cerebral I/R damage. The findings from our study demonstrated that the levels of TNF-α and IL-6 in rats of TEAS group were obviously reduced compared to MCAO/R group, indicating that TEAS therapy effectively mitigated neuroinflammation to alleviate the ischemic damage caused by stroke via stimulating the specific acupoints. Therefore, inhibiting excessive inflammatory response is an important mechanism for TEAS to treat ischemic stroke.

Apoptosis, also known as programmed cell death, is characterized by cellular shrinkage, condensation of cytoplasm, degradation of DNA, and fragmentation (Elmore, 2007). More and more evidence has shown that inhibiting apoptosis can significantly reduce cell injury following cerebral I/R damage (Zhang et al., 2018). Chen et al. demonstrated that administering EA therapy on the Quchi and Zusanli acupoints enhanced the proportion of Bcl-2/Bax (an indicator of anti-apoptosis ability) and improved neural function following ischemic stroke (Chen et al., 2012). Accordingly, the results in our study indicated that TEAS had the ability to enhance the levels of Bcl-2 and suppress the expression of Bax when compared to MCAO/R group, suggesting that TEAS was able to exert inhibitory effect on neuronal cell apoptosis in ischemic stroke. To sum up, blocking cell death is a crucial cause for TEAS to play a protective role in the treatment of ischemic stroke.

Pyroptosis, in contrast to apoptosis, is essentially characterized by cellular perforation and the generation of intracellular pro-inflammatory factors due to caspase activation, resulting in inflammatory cell death (Cookson and Brennan, 2001). As for the relationship between NLRP3 inflammasome and pyroptosis, the NLRP3 inflammasome triggers caspase-1 activation, resulting in the transformation of IL-1β precursor (pro-IL-1β) and pro-IL-18 into IL-1β and IL-18 (Chumboatong et al., 2022). Caspase-1 activation also results in the cleavage of GSDMD, generating oligomers of pore-forming proteins known as N-terminal fragment oligomers (GSDMD-N) in the cytoplasm. These -∧#GSDMD-N proteins then form pores of 10–14 nm in the cytomembrane, leading to the release of IL-1β and IL-18 into the intercellular space, ultimately triggering pyroptosis (Ding et al., 2016). Hence, suppressing pyroptosis following cerebral ischemia can mitigate brain injury and provide a safeguarding impact (She et al., 2019; Ran et al., 2021). According to our findings, TEAS was found to reduce the levels of NLRP3, cleaved caspase-1/pro caspase-1, IL-1β, IL-18, and GSDMD, thereby suppressing pyroptosis after brain I/R in comparison to MCAO/R group. In summary, TEAS exerts a protective role by inhibiting pyroptosis of neuronal cells following brain ischemia.

Microglia, which are immune cells that are always present in central nervous system (CNS), act as the initial line of defense for the immune system (Colonna and Butovsky, 2017). Microglia activation is regarded as the primary stimulus for immune response in brain tissue (Longa et al., 1989). After an acute ischemic stroke, the activation of microglia can promptly induce an elevation in the levels of pro-inflammatory factors such as TNF-α and IL-1β, resulting in inflammation, neuronal demise, and brain damage (Yu et al., 2021). Our findings indicated that the level of Iba1 (a marker of microglial activation) was reduced in TEAS group in comparison with MCAO/R group, and the TLR4 activator (LPS) significantly reversed the protective effect of TEAS, indicating that TEAS treatment can exert a protective effect by suppressing microglial activation via regulating the TLR4 pathway.

Toll-like receptor 4, as one of the most researched TLRs, was significantly activated after cerebral ischemia, and the inhibition of TLR4 could effectively reduce inflammation following ischemic stroke (Andresen et al., 2016). The activation of TLR4 results in activation of the adaptor protein MyD88, which facilitates the movement of NF-κB from the cytoplasm to the nucleus, initiating the inflammatory reactions (Hua et al., 2009). The inflammatory response regulated by TLR4/MyD88/NF-κB is crucial in causing damage to the brain during I/R (Tao et al., 2016). Furthermore, the activated NF-κB pathway exerts vital significance in inflammation and also has a crucial impact on the regulation of apoptosis (Hoffmann and Baltimore, 2006). In ischemic stroke, the TLR4/NF-κB pathway has the ability to trigger the activation of NLRP3 inflammasome, which in turn regulates pyroptosis (El-Sisi et al., 2021). Hence, blocking the TLR4/MyD88/NF-κB pathway is a crucial approach to diminish neuronal cell inflammation, while also suppressing apoptosis and pyroptosis levels, ultimately easing the impact of ischemic injury in cases of ischemic stroke. Research has confirmed that EA at Quchi (LI11) and Zusanli (ST36) acupoints could alleviate inflammation and reduce cerebral I/R damage via TLR4/NF-κB pathway (Lan et al., 2013). In our research, the TLR4/MyD88/NF-κB pathway became active following brain I/R injury. Additionally, there was a noticeable rise in neuronal inflammation, apoptosis, and pyroptosis. This was evident through increased levels of TNF-α and IL-6 expression, as well as elevated Bax expression and reduced Bcl-2 expression. Furthermore, the levels of NLRP3, cleaved caspase-1/pro caspase-1, IL-1β, IL-18, and GSDMD were increased, as well as there was a notable activation of microglia cells. Following TEAS therapy, there was a decrease in neuronal cell inflammation, reduced levels of apoptosis and pyroptosis, and a reduction in microglia activation. Moreover, LPS, which is a TLR4 agonist, counteracted the impacts of TEAS, indicating that the TLR4/MyD88/NF-κB pathway exerts vital effects on the defensive mechanisms of TEAS against ischemic stroke. To summarize, the use of TEAS can suppress the activation of microglia and levels of inflammation, apoptosis, and pyroptosis in neuronal cells following ischemic stroke via suppressing the TLR4/MyD88/NF-κB pathway.

There were two main limitations in this research. First, our study mainly focused on the protective roles of TEAS on acute ischemic stroke, without exploring the different effects of TEAS at various acupoints. In our upcoming research, we will investigate the defensive benefits of TEAS on additional acupuncture points. Second, microglia play a contrasting role of harm or benefits on the CNS, which is associated with the two separate characteristics of M1 and M2 microglia (Hu et al., 2012). The mechanism by which TEAS control the transitions between M1 and M2 phenotypes is still not understood. In upcoming research, we aim to analyze the levels of M1 and M2 microglia indicators to gain a deeper understanding of the impact of TEAS therapy on microglia polarization during the initial stage of ischemic stroke.

## Conclusion

To sum up, the administration of TEAS can enhance neurological function, decrease the size of infarction, and alleviate the pathological damage of hippocampus in rats following ischemic stroke. Furthermore, TEAS therapy has the ability to reduce the inflammatory reaction following I/R, hinder the apoptosis and pyroptosis of neuronal cells, and restrain the activation of microglia by suppressing the TLR4/MyD88/NF-κB pathway. In this present study, the effectiveness of TEAS on ischemic stroke is confirmed, and therapeutic mechanisms of TEAS in the acute phase of ischemic stroke is investigated, offering a promising and feasible treatment option for patients following ischemic stroke.

## Data availability statement

The raw data supporting the conclusions of this article will be made available by the authors, without undue reservation.

## Ethics statement

The animal study was approved by the Laboratory Animal Ethical and Welfare Committee of Hebei Medical University. The study was conducted in accordance with the local legislation and institutional requirements.

## Author contributions

LW: Conceptualization, Data curation, Formal Analysis, Writing−original draft. ZT: Formal Analysis, Software, Writing−original draft. LS: Conceptualization, Data curation, Methodology, Writing−original draft. FD: Data curation, Formal Analysis, Writing−original draft. GX: Data curation, Investigation, Methodology, Writing−original draft. FZ: Conceptualization, Formal Analysis, Funding acquisition, Supervision, Writing−original draft, Writing−review and editing.
