# Supplementary material for: Transcutaneous electrical acupoint stimulation alleviates cerebral ischemic injury through the TLR4/MyD88/NF-κ B pathway
Source: Front Cell Neurosci. 2024 Jan 11;17:1343842. doi: 10.3389/fncel.2023.1343842 (PMC10808520; doi:10.3389/fncel.2023.1343842)
Supplement: Supplementary file 1 [file Data_Sheet_1.docx]

Supplementary Material

# Supplementary Figures


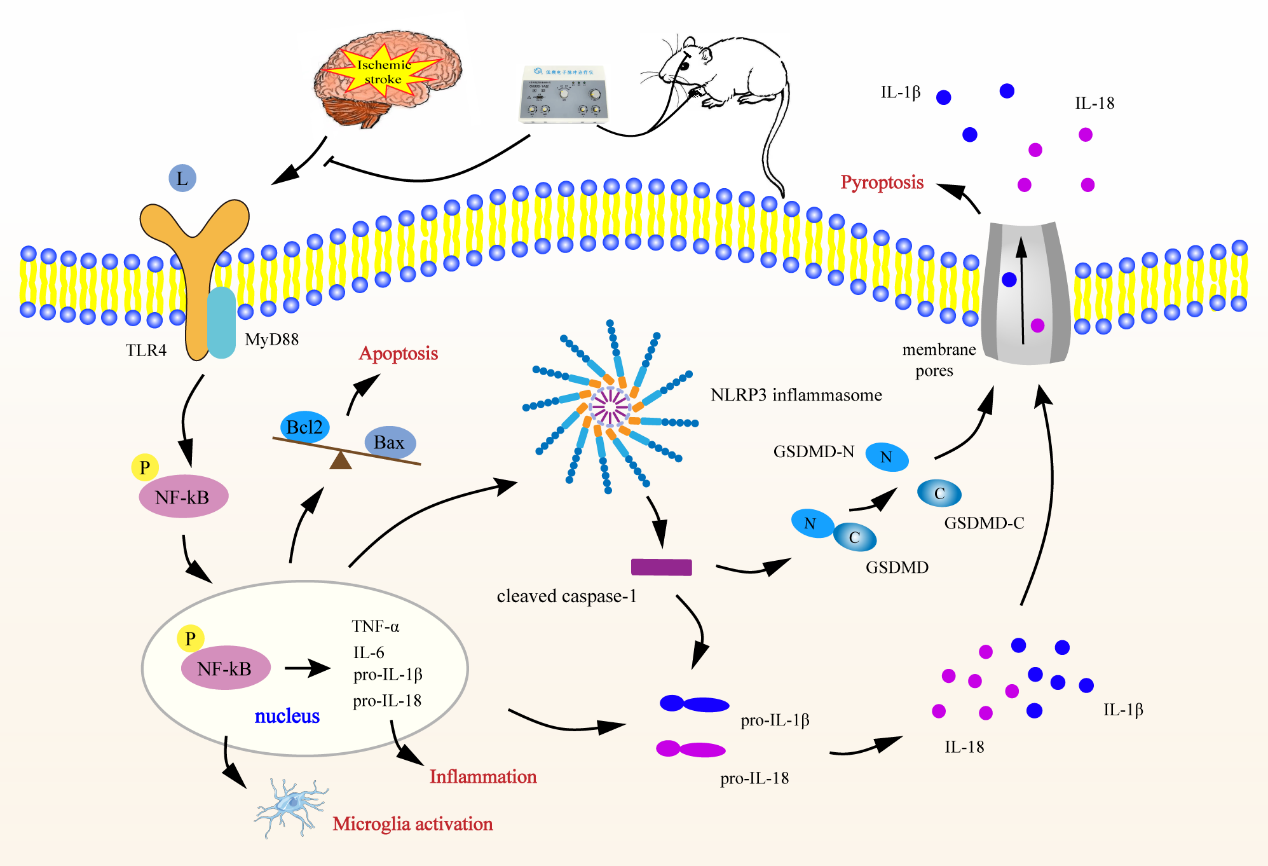


**Supplementary Figure 1.** The schematic diagram of mechanisms of TEAS against ischemic injury.

# Supplementary Materials

# 2.1 2,3,5-Triphenyltetrazolium Chloride (TTC) Staining

After 72 hours of reperfusion, the rats (n=5/group) were promptly sacrificed and then frozen at -20°C for a duration of 15 minutes. Then cut the brain into six slices with a thickness of 2mm per slice. The slices were immersed in a 2% TTC solution at a temperature of 37℃ for a duration of 30 minutes. Subsequently, they were transferred to a 4% paraformaldehyde buffer and left there for a period of 24-48 hours. A picture was taken of the slices and the volume of infarction was evaluated using Image J. The infarct volume ratio (%) is determined by adding up the volumes of infarcts in the six slices (the total volume of cerebral infarcts) and dividing it by the total brain volume, then multiplying by 100%.

# 2.2 Western blot (WB) Analysis

WB was used to detect the levels of proteins in the TLR4/MyD88/NF-kB pathway and proteins related to inflammation, apoptosis, and pyroptosis. Rats (n=5 per group) were sacrificed and the target protein was detected. The injured hippocampus was collected and hippocampal tissues were lysed. The same quantity of proteins (20µg) were placed into SDS-PAGE gels for electrophoresis and then transferred to PVDF membranes (Roche, Mannheim, Germany). Following a 2-hour incubation at 37℃ in a 5% solution of bovine serum albumin, the membrane was then underwent incubation for 12 hours at 4℃ with primary antibodies. Following the TBST wash, the membranes were exposed to goat anti-mouse IgM (1:5000, SA00001-1, proteintech) or anti-rabbit IgG (1:5000 SA00001-2, proteintech) antibodies for 1 hour at room temperature. Image J was used to calculate the optical density value for each strip.

# 2.3 Immunohistochemistry (IHC) Analysis

Five rats per group were euthanized without pain, and their brains were fixed in 4% paraformaldehyde for 24 hours. Paraffin-embedded tissues were prepared. The paraffin-embedded sections underwent dewaxing, hydration, and a 20-minute incubation with 3% H2O2. After being sealed with goat serum for 20 minutes, the slides underwent incubation for 12 hours at 4℃ with the primary antibody TLR4 (1:100, AF7017, Affinity). Following a TBST wash, the sections were treated with goat anti-rabbit IgG for 60 mins our at ambient temperature and examined using 3,3'-diaminobenzidine chromogen. In the end, the slices were dried out and attached to slides. The hippocampus was photographed with magnification of 400 times. Image J was used to assess the expression of TLR4 in all groups.

# 2.4 Immunofluorescence (IF) Staining

Rats (n=5 per group) were decapitated painlessly. Immediately, the brain was preserved using a 4% solution of paraformaldehyde. Following a 24-hour fixation period, the brains were encased in paraffin blocks. Coronal sections of 4µm thickness were obtained by cutting the paraffin blocks. Sheep serum that is typical was utilized to obstruct the binding of non-specific antigens for a duration of 60 minutes. The sections were treated with Iba1 antibodies (1:1000, 013-26471, Wako) and incubated for 12 hours at 4℃. Then the sections were treated with a red fluorescent secondary antibody (1:400, SA00007-2, proteintech) in a dark environment for 2 hours at ambient temperature, followed by three washes with PBS. The nuclei were stained with DAPI, specifically 40, 6-diamidine-2-phenylindole. Fluorescence microscope (Olympus, Japan) was used to capture and photograph cells in the hippocampus, magnified at 200 times. Image J was used for measuring the mean fluorescence intensity.
